# Impact of MAPK Pathway Activation in BRAF^V600^ Melanoma on T Cell and Dendritic Cell Function

**DOI:** 10.3389/fimmu.2013.00346

**Published:** 2013-10-28

**Authors:** Patrick A. Ott, Nina Bhardwaj

**Affiliations:** ^1^Dana Farber Cancer Institute, Harvard Medical School, Boston, MA, USA; ^2^Tisch Cancer Institute, Mount Sinai School of Medicine, New York, NY, USA

**Keywords:** melanoma, dendritic cell, T cell, BRAF, MEK, immunotherapy, kinase inhibitor

## Abstract

Constitutive upregulation of the MAPK pathway by a BRAF^V600^ mutation occurs in about half of melanomas. This leads to increased oncogenic properties such as tumor cell invasion, metastatic potential, and resistance to apoptosis. Blockade of the MAPK pathway with highly specific kinase inhibitors induces unprecedented tumor response rates in patients with advanced BRAF^V600^ mutant melanoma. Immune checkpoint blockade with monoclonal antibodies targeting cytotoxic T-lymphocyte antigen 4 and programed death-1/PD-L1 has also demonstrated striking anti-tumor activity in patients with advanced melanoma. Tumor responses are likely limited by multiple additional layers of immune suppression in the tumor microenvironment. There is emerging preclinical and clinical evidence suggesting that MAPK inhibition has a beneficial effect on the immunosuppressive tumor microenvironment, providing a strong rationale for combined immunotherapy and MAPK pathway inhibition in melanoma. The T cell response has been the main focus in the studies reported to date. Since dendritic cells (DCs) are important in the induction of tumor-specific T cell responses, the impact of MAPK pathway activation in melanoma on DC function is critical for the melanoma directed immune response. BRAF^V600E^ melanoma cells modulate DCs through the MAPK pathway because its blockade in melanoma cells can reverse suppression of DC function. As both MEK/BRAF inhibition and immune checkpoint blockade have recently taken center stage in the treatment of melanoma, a deeper understanding of how MAPK pathway inhibition affects the tumor immune response is needed.

## Introduction

Melanoma incidence rates have been increasing for at least 30 years. It is estimated that 76,690 individuals will be diagnosed in 2013 (1). The disease is usually curable when detected in its early stages (thin primary tumor, no lymph node involvement). For patients with unresectable or metastatic melanoma, recently emerged novel systemic treatment modalities such as Cytotoxic T-Lymphocyte Antigen 4 (CTLA-4) and Programed Death-1 (PD-1)/PD-L1 blockade as well as BRAF and MEK inhibition have expanded the spectrum of therapeutic options (2–13). The successes with immune checkpoint blocking antibodies in the treatment of patients with metastatic melanoma, with reported response rates of up to 50% are remarkable. Both CTLA-4 and PD-1/PD-L1 blockade can induce long lasting tumor responses in the absence of vaccination, suggesting that endogenous tumor-specific T cells exist in a substantial proportion of patients and that these T cells, once uncoupled from the inhibitory effect mediated by CTLA-4 and/or PD-1/PD-L1 can mediate effective tumor cell lysis (2, 3, 12–15). Multiple other immune suppressive mechanisms are at work in the tumor environment, including additional inhibitory molecules such as Tim-3 (16) and LAG-3 (17), regulatory T cells, myeloid derived suppressor cells (18), and soluble immunosuppressive mediators such as IDO (indoleamine 2,3-dioxygenase), arginase, prostaglandin E2 (PGE2), IL-6, IL-10, VEGF, TGF-β along with other suppressive cytokines and chemokines. Given the multitude of suppressive mechanisms, it is remarkable that a relatively high proportion of patients can achieve objective tumor responses by blockade of a single pathway, such as PD-1/PD-L1 or CTLA-4.

Approximately half of melanomas harbor a somatic point mutation of the BRAF oncogene at codon 600 (V600E and V600K). This mutation results in constitutive activation of the MAPK pathway and increased oncogenic behavior mediated through a variety of mechanisms such as increased apoptosis, invasiveness, and metastatic potential. The MAPK pathway is an important therapeutic target in melanoma: BRAF, MEK, and combined BRAF/MEK inhibition with small molecule kinase inhibitors are successful treatment strategies in patients with BRAF mutant metastatic melanoma (6–10). However, resistance to these treatments develops almost universally, limiting the median duration of treatment responses to 6–9 months. Investigation of resistance mechanisms and potential strategies to overcome resistance is a very active area of research; a number of different mechanisms have been identified, including the reactivation of MAPK signaling by other pathways (19–24).

**Figure 1:**
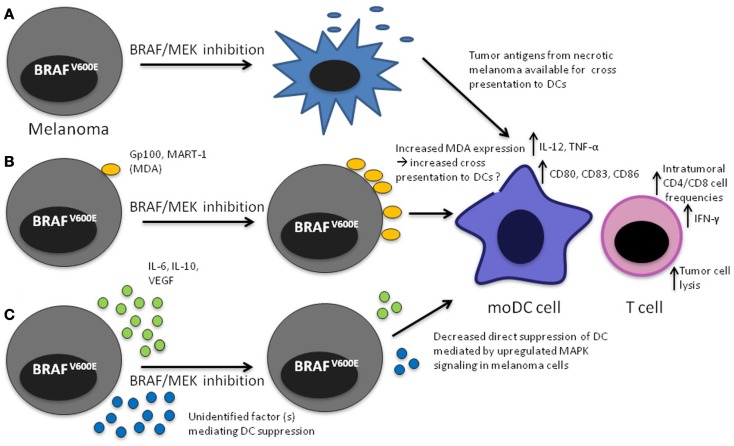
**Mechanisms that may lead to increased DC function upon MAPK pathway blockade in the tumor microenvironment**. **(A)** Apoptosis/necrosis of melanoma cells results in release of tumor antigens that will presumably be available to DCs for cross presentation; **(B)** Increased expression of MDA through direct effect of MAPK pathway inhibition, potentially making them available to DCs for cross presentation, **(C)** decreased direct inhibition of DCs leading to increased IL-12 and TNF-α production.

Given the treatment successes with both kinase inhibition and immune checkpoint blockade in melanoma, there is considerable interest in combinatorial approaches. The promise is to combine the response durability that is characteristic for patients responding to immunotherapy with the high response rate seen with BRAF inhibition. The scientific rationale for such strategies is based on the interplay of the MAPK pathway and the tumor immune response in the microenvironment. Activation of signaling pathways in tumor cells have long been implicated in promoting suppressive immune networks in the tumor environment (25, 26). There is emerging evidence of a link between the MAPK pathway in melanoma and the tumor immune response. Preclinical and clinical observations indicate that inhibition of the MAPK pathway may have a favorable effect on the melanoma-specific immune response on the level of T cells, tumor cells, stromal cells, and dendritic cells (DCs) (Table 1; Figure 1).

**Table 1:** **Effects of MAPK inhibition on immune function and melanoma**.

Study type	Model	Immune cell type studied	Effect of MAPK inhibition	
			Immune cells	Melanoma cells
*In vitro* (human) (38)	Monocyte-derived moDC co-cultured with BRAF^V600E^ mutant and WT melanoma cell lines DC maturation with Poly-ICLC	DCs	Restored IL-12 and TNF-α production by DCs exposed to BRAF mutant melanoma cells treated with MEK and BRAF inhibition	No consistent suppression of cytokine production observed
*In vitro* (human) (36)	Monocyte-derived moDC cultured with supernatants of BRAF^V600E^ mutant melanoma cell lines DC maturation with LPS	DCs	Restored IL-12 and TNF-α production by DCs exposed to supernatants of melanoma cells treated with BRAF^V600E^ – specific RNAi	Suppression of IL-6, IL-10, and VEGF secretion
*In vitro* (human) (27)	BRAF^V600E^ mutant and WT melanoma cell lines treated with MEK and BRAF inhibition. Melanoma cells cultured with TCR-transgenic CTL specific for gp100, MART-1	CTL	Increased IFN-γ production by melanoma-specific CTL cultured with BRAF^V600E^ melanoma upon MEK and BRAF inhibition	Increased expression of MDA
*In vitro* (human) (42)	Mixed lymphocyte reaction with DCs, PBMCs, and T cells	DCs, T cells	Suppressed T cell activation by DCs exposed to melanoma overexpressing CD200; effect abrogated by CD200 knockdown with shRNA	Not assessed
Mouse adoptive T cell transfer (35)	BRAF^V600E^-driven murine model of SM1 melanoma Adoptive transfer of C57BL/6 mice with TCR-transgenic lymphocytes	OVA and pmel-1 TCR-transgenic lymphocytes	No effect on expansion, distribution, or tumor accumulation of adoptively transferred T cells Increased T cell functionality (IFN-γ production, intrinsic tumor cell lysis)	No effect on gp100 expression on SM1 cells Increased tumor response with BRAF inhibition + adoptive T cell transfer
Mouse adoptive T cell transfer (32)	Xenograft with gp100 expressing melanoma cell lines. Adoptive transfer of C57BL/6 mice with TCR-transgenic gp100-specific pmel-1 T cells	Pmel-1 TCR-transgenic T cells	Enhanced infiltration of BRAF mutant, but not BRAF WT tumors with adoptively transferred T cells Increased VEGF production in tumors	Increased tumor response with BRAF inhibition + adoptive T cell transfer
Melanoma patients (34)		Intra-tumoral CD4 cells, CD8 cells, CD20 cells, Granzyme B, CD1a^+^ DC	Increased CD4 and CD8 cell frequencies in post-treatment tumor specimens	Objective tumor responses on CT imaging
			Correlation between increased tumor CD8 infiltration and decreased tumor size and increase in tumor necrosis	
			Occasional CD1 DCs present in post-treatment biopsies in 2 patients	
Melanoma patients (31)		Intra-tumoral CD4^+^ cells, CD8^+^ cells, IL-6, IL-8, IL-10, TGF-β, granzyme B, perforin, Tim-3, PD-1, PD-L1	Increased CD8^+^ cell frequencies No effect on CD4 cells Decreased IL-6 and IL-8 production Increased expression of Tim-3, PD-1, PD-L1 No effect on IL-10, TGF-β	Objective tumor responses on CT imaging Increased expression of MDA (MART-1, gp100, TYRP-1, TYRP-2)

## BRAF and MEK Inhibition in Melanoma Cell Lines Leads to Upregulation of Tumor Antigens and Increased Recognition by Melanoma-Specific T Cells *In vitro*

In melanoma cell lines, MEK and BRAF inhibition leads to increased expression of melanoma differentiation antigens (MDAs) such as gp100, MART-1, and tyrosinase on the mRNA and protein levels (27–29). The underlying mechanism of oncogenic BRAF-regulated MDA expression is unclear. It has been suggested that oncogenic BRAF suppresses MDA expression through microphthalmia-associated transcription factor. However additional pathways are likely involved and may account for the heterogeneity of MDA induction observed across different cell lines including mutant and wild-type cell lines (30). Increased expression of gp100 and MART-1 leads to improved antigen recognition by T cells as measured by IFN-γ production (27). Upregulation of gp100 and MART-1 was seen in both BRAF mutant and WT melanoma cell lines. BRAF inhibition did not negatively impact lymphocyte function, whereas MEK inhibition negatively affected T cell proliferative potential, viability, and IFN-γ production. These data were recently confirmed *in vivo* in patients with metastatic melanoma (31). Increased MART, TYRP-1, TYRP-2, and gp100 expression was found in metastatic melanoma specimens obtained from patients after treatment with BRAF and/or MEK inhibition. Interestingly, melanoma antigen expression in metastatic tumors was decreased at the time of tumor progression in patients treated with a BRAF inhibitor and partially restored upon initiation of dual MEK and BRAF blockade.

## Increased Frequency of Tumor Infiltrating Lymphocytes after BRAF Inhibition

In an adoptive T cell transfer (ACT) model, frequencies of gp100 specific luciferase expressing pmel-1 T cells were markedly increased in gp100 expressing melanoma lesions after treatment with vemurafenib (32) and this was associated with improved tumor response compared to either vemurafenib or ACT alone. This observation was specific to BRAF mutant tumors and independent of BRAF inhibition-mediated upregulation of MDA. In this model, the increased intra-tumoral T cell frequencies were attributed to decreased VEGF in the tumor. It was previously shown that VEGF/VEGFR-2 inhibition can upregulate endothelial adhesion molecules in tumor vessels, which can in turn increase the infiltration of leukocytes in tumors (33). Wilmott et al. confirmed the observations of increased intra-tumoral T cell frequencies in melanoma patients who were treated with a BRAF inhibitor (34). Increased frequencies of CD4 and CD8 cells were seen in both intra-tumoral and peritumoral regions of metastatic tumor specimens obtained between 3 and 15 days after treatment initiation. The increase in lymphocyte numbers inversely correlated with tumor size, but not with clinical objective responses. Notably, intra-tumoral lymphocyte frequencies returned to pre-treatment levels at the time of tumor progression. Similar increases in tumor infiltration by CD8 cells (but not CD4 cells) and decrease upon tumor progression in melanoma patients treated with BRAF or dual BRAF/MEK inhibition was reported by Frederick et al. (31).

## MAPK Inhibition Affects T Cell Functionality and Secretion of Immunosuppressive Cytokines in the Tumor Microenvironment

In an ACT model using the murine BRAF^V600E^ mutant melanoma SM1 and transgenic T cells recognizing gp100 and ovalbumin (OVA), combined ACT and vemurafenib induced superior anti-SM1 tumor immune responses compared to either of the therapies alone. In this study, no difference in frequencies of adoptively transferred T cells was observed in tumors, lymph nodes, or spleen as assessed *ex vivo* by flow cytometry and immunofluorescence imaging and *in vivo* by tracking of the firefly luciferase transgene-labeled T cells using bioluminescence imaging when mice were treated with vemurafenib in addition to ACT. However, adoptively transferred T cells exhibited increased functionality as measured by IFN-γ production and their ability to lyse tumor cells (35) in mice treated with ACT and vemurafenib.

## Cross-Talk between the MAPK Pathway in BRAF Mutant Melanoma and DCs

Sumimoto et al. demonstrated that BRAF^V600E^ mutant cell lines can produce immunosuppressive cytokines such as VEGF, IL-6, and IL-10 and that MEK inhibition with U0126 and BRAF inhibition using BRAF^V600E^ specific RNAi suppressed secretion of these cytokines. IL-12 and TNF-α production by DCs exposed to supernatant from the BRAF mutant A375 melanoma cell line prior to maturation by LPS was suppressed (36). This inhibitory effect was mediated by IL-6, IL-10, and VEGF and could be partially reversed by pre-treatment of the melanoma cells with BRAF^V600E^ specific RNAi, indicating that constitutive activation of the MAPK pathway in melanoma cells may lead to compromised DC function and that this immune evasion may be overcome by MAPK inhibition. In a separate study, IL-10 expression in the melanoma line A375 was found to be induced by TGF-β, an effect that was mediated by cross-talk between the Smad, PI3K/AKT, and MAPK pathways (37).

We recently explored a potential link between constitutive MAPK pathway upregulation driven by a BRAF^V600^ mutation and DC function using a human melanoma-DC co-culture system (38). BRAF^V600E^ mutant and wild-type melanoma cell lines were treated for 24 h with the MEK inhibitor U0126, the BRAF inhibitor vemurafenib, or respective controls (U0124 or DMSO). After removal of supernatant, monocyte-derived immature DC from healthy donors were added, cultured for 24 h and then stimulated with poly-ICLC. Poly-ICLC was chosen as the DC maturation stimulus because it induces the secretion of proinflammatory cytokines in the absence of IL-10 and is a potent TLR3 and MDA5 agonist (39). It has been widely used as a cancer vaccine adjuvant in clinical trials. We found that IL-12 and TNF-α production by DCs was inhibited when DCs were exposed to melanoma cells treated with vehicle control. Notably, the secretion of both cytokines could be partially or completely restored with both MEK and BRAF inhibition in BRAF^V600E^ mutant, but not wild-type cell lines. Furthermore, CD80, CD83, and CD86 expression on DC was decreased upon co-culture with melanoma cells and could be partially restored with BRAF inhibition in BRAF^V600E^ mutant melanoma cell lines. The inhibition of IL-12 and TNF-α secretion by DCs was not cell-contact dependent. In contrast to the study by Sumimoto, a soluble factor responsible for mediating the suppressive effect could not be identified in our investigations. It is possible that continuous local production of small amounts of soluble mediators by melanoma cells in close proximity to DCs accounts for the inhibitory effect observed in the melanoma cell/DC co-culture experiments in our study.

CD200, a type I membrane-associated glycoprotein and member of the immunoglobulin superfamily is highly expressed on melanoma cells and was found to be regulated by ERK activation (40). CD200 mRNA expression levels were found to be positively correlated with tumor progression. Moreover, MEK inhibition with U0126 and knockdown of mutant BRAF resulted in reduced expression of CD200 mRNA in melanoma cell lines. Of note, through interaction with the CD200 receptor, which is expressed on macrophages and DC, CD200 mediates an inhibitory signal (41). In mixed lymphocyte reactions with T cells, DCs, and melanoma cells, T cells produced larger amounts of IL-2 when CD200 in melanoma cells was knocked down with shRNA specifically targeting the CD200 ligand (42). These data suggest a link between MAPK/ERK activation in melanoma and the ability of DCs to activate T cells.

## Direct Impact of MAPK Inhibition on DCs

Since there is a strong clinical interest in combined immunotherapy and BRAF/MEK inhibition in melanoma, the direct impact of MAPK pathway inhibition on immune cells is of great interest. BRAF inhibition, even at high concentrations, does not appear to directly compromise T cell function, and there is emerging data showing that low doses of RAF inhibition may even enhance T cell activation (43, 44). Furthermore, frequencies of DCs, monocytes, T cells, B cells, NK cells, and regulatory T cells in peripheral blood from metastatic melanoma patients were not affected by BRAF inhibition (45).

There is some controversy about the direct impact of signaling through the MAPK pathway on DC maturation. In LPS and TNF-α-matured DCs, MEK inhibition leads to upregulation of co-stimulation molecules, increased IL-12 secretion and enhanced ability to activate T cells (46), whereas activation of ERK in DCs leads to immune suppression, mediated by TGF-β and Treg cells (47). Only minimal or no effect of MEK inhibition on DC function was shown in other studies (48–51). Differences in the maturation stimuli may account for some of the inconsistencies observed in these investigations.

In monocyte-derived DC from healthy donors, MEK inhibition lead to reduced IL-12 and TNF-α secretion, whereas BRAF inhibition had no effect on cytokine production over a wide range of doses (38). The expression of CD40, CD80, CD83, and MHCI was also reduced by direct MEK inhibition, whereas it was unaffected by BRAF inhibition. In addition, DC viability was reduced with MEK, but not BRAF inhibition and the ability of DCs to induce T cell proliferation in an MLR was reduced with MEK, but not BRAF inhibition. The impact of MEK inhibitors currently used in the clinic on APC function *in vivo* remains to be determined.

## MAPK Inhibition may Enhance DC Function in the Tumor Microenvironment by Several Mechanisms

### Restoration of DC function compromised by melanoma cells

Our studies and earlier investigations by Sumimoto suggest that suppression of IL-12 and TNF-α production by DCs in the tumor microenvironment of a BRAF^V600^ mutant melanoma is mediated at least partially by constitutive activation of the MAPK pathway. These data also indicate that BRAF and MEK inhibition, by blocking the MAPK pathway in melanoma cells and thereby restoring IL-12 and TNF-α production in DCs, leads to improved DC function, presumably leading to better activation of melanoma-specific T cells. Notably in our studies there was none or only minimal apoptosis in BRAF^V600E^ mutant and WT melanoma cell lines after 48 h of MEK or BRAF inhibition. This is consistent with prior studies showing an anti-proliferative effect, rather than apoptosis, during the first few days of treatment with these kinase inhibitors (52, 53). It is therefore unlikely that the reversal of compromised DC function mediated by melanoma cells in the *in vitro* experiments is mediated by melanoma cell death. Similarly, in the experiments by Sumimoto, no significant cell death was observed after treatment of the melanoma line A375 with the MEK inhibitor U0126, indicating that decreased IL-10, IL-6, and VEGF production was a direct effect of MAPK pathway inhibition rather than mere death of the melanoma cells. Taken together, these data suggest that MAPK pathway activation in BRAF^V600^ mutant melanoma cells has a direct suppressive effect on the capacity of DC to activate T cells.

### Increased cross presentation of overexpressed melanoma differentiation antigens by DCs in the tumor or lymph node?

Inhibition of the MAPK pathway in BRAF mutant melanoma leads to increased expression of MDAs (gp100, Mart-1, Tyrp-1, and Tyrp-2), resulting in improved antigen-specific recognition by gp100 and MART-1 specific TCR-transgenic CTL as measured by increased IFN-γ production *in vitro* (27). In patients with BRAF^V600^ mutant metastatic melanoma, MART-1 expression was upregulated in metastatic tumors after treatment with BRAF inhibition. Increased infiltration of metastatic tumors with both CD4 and CD8 cells in one study, and of CD8 cells, but not CD4 cells in another study was observed after treatment with BRAF inhibition. A correlation between intra-tumoral infiltration with CD8 cells and tumor necrosis was found in post-treatment biopsies in one study (34). In addition to the direct effect on CTL function shown *in vitro*, it is possible that MDA overexpression on melanoma cells in the tumor *in vivo* may lead to increased cross presentation of these antigens to DCs and thus further enhance the tumor-specific T cell response.

### Increased cross presentation of tumor antigens derived from apoptotic tumor cells after MAPK inhibition

As outlined above, in short-term (48–72 h) *in vitro* experiments using BRAF mutant melanoma cell lines, apoptosis or necrosis does not have a significant role in mediating the effects of melanoma MAPK pathway inhibition on DCs. BRAF inhibition does however eventually induce apoptosis and necrosis as evident by the fact that tumors shrink markedly in the majority of patients. Tumor necrosis/apoptosis likely leads to the release of antigens, which may be available for DCs either residing in the tumor or in draining lymph nodes to be taken up, processed, and cross-presented to T cells. Cross presentation may be one of the mechanisms mediating the synergy observed with ACT and BRAF inhibition in melanoma mouse models, although no direct evidence was provided in the reported studies (32, 35).

## Conclusion

Constitutive upregulation of the MAPK pathway in BRAF^V600^ mutant melanoma appears to directly impact DC function as evident by partial restoration of IL-12 and TNF-α secretion upon treatment of melanoma cells with MEK or BRAF inhibition. These effects have so far been shown only *in vitro*. The beneficial effects of MAPK blockade on the tumor immune microenvironment shown *in vivo* in mouse models and melanoma patients argue for a broader impact of these treatments on the tumor-specific immune response, including increased T cell frequencies and improved function and changes in cytokine secretion patterns. Several mechanisms that may account for the improved immune response have been described such as increased MDA expression on melanoma cells and decreased intra-tumoral VEGF production, others remain speculative, such as increased cross presentation to DCs resulting from BRAF/MEK inhibition-mediated necrosis/apoptosis of melanoma cells. These observations reinforce the rationale for clinical trials assessing MEK/BRAF inhibition and immunotherapy in combination in patients with melanoma. Further studies are needed to delineate the phenotype and function of DCs in patients treated with BRAF/MEK inhibition. Because of superior efficacy and potentially improved tolerability, combined BRAF-MEK inhibition will likely replace BRAF inhibitor monotherapy. Defining the impact of both BRAF and MEK inhibition on the immune response will therefore be critical.

## Conflict of Interest Statement

The authors declare that the research was conducted in the absence of any commercial or financial relationships that could be construed as a potential conflict of interest.
